# Editorial: The molecular mechanisms and potential drug targets for remyelination and neuroregeneration

**DOI:** 10.3389/fnmol.2023.1332594

**Published:** 2023-11-17

**Authors:** Aleksandra Rutkowska, Maria Velasco-Estevez, Sinead A. O'Sullivan

**Affiliations:** ^1^Brain Diseases Centre, Medical University of Gdansk, Gdańsk, Poland; ^2^Department of Anatomy and Neurobiology, Medical University of Gdansk, Gdańsk, Poland; ^3^H12O-CNIO Hematological Malignancies Group, Clinical Research Unit, Centro Nacional de Investigaciones Oncologicas (CNIO), Madrid, Spain; ^4^German Centre for Neurodegenerative Diseases, Helmholtz Association of German Research Centers (HZ), Bonn, Germany

**Keywords:** multiple sclerosis, remyelination, disease-modifying therapies, regeneration, neurodegeneration

Multiple sclerosis (MS), a chronic, progressive, and often debilitating neurological disorder, has long remained a formidable challenge in the field of neurology. Its etiology, involving complex interactions between genetic, environmental, and lifestyle factors, has confounded researchers. As an ailment that affects an estimated 2.3 to 2.5 million people worldwide, the importance of understanding its pathogenesis and finding effective treatments cannot be overstated. This Research Topic is a beacon of progress in the relentless quest to promote remyelination and neuroregeneration in MS and related demyelinating and neurodegenerative pathologies.

Recent years have witnessed significant strides in the development of disease-modifying therapies (DMTs) aimed at managing primarily the relapsing-remitting form of MS. Currently, there are 23 FDA-approved DMTs available, which have substantially improved the quality of life for many MS patients. However, these therapies are not without drawbacks, with some having potentially serious side effects. Moreover, their principal mechanism of action predominantly targets the peripheral immune system, mitigating neuroinflammatory signaling or limiting peripheral lymphocyte infiltration into the central nervous system (CNS), while offering limited effects on neuroregeneration and remyelination. A crucial factor that underscores the urgency of innovative treatments is the consistent loss of brain matter from the outset of the disease, progressing at equal rates across different MS subtypes. Therefore, identifying druggable targets capable of protecting against neurodegeneration and stimulating neuroregeneration and remyelination is of paramount importance.

This Research Topic unites six papers, each presenting distinct perspectives and emerging methods to tackle the obstacles related to remyelination and neuroregeneration in MS and other demyelinating diseases. Let us explore the salient features of each paper and the cumulative impact of this collective effort.

Acheta et al. delve into the role of CC2D1B in developmental myelination within the central nervous system. This paper elucidates the significance of CC2D1B in myelin formation, notably in optic nerves. It underscores the intricate interactions of transcription factors that orchestrate myelination. These findings have the potential to provide new avenues for the development of therapeutic interventions in the remyelination process.

The next paper by Kutryb-Zajac et al. focuses on the altered distribution and activity of adenosine deaminase (ADA) isoenzymes in the context of MS. ADA plays a vital role in regulating adenosine levels and modulating neuroinflammation. This paper sheds light on the complexities of ADA regulation and its impact on the pathophysiology of MS. These insights offer promising avenues for further research into potential therapeutic interventions.

Ferret-Sena et al. explores the potential of modulating peroxisome proliferator-activated receptors (PPARs) and CD36 gene expression through fingolimod treatment. Fingolimod, an immunomodulatory drug, is suggested here to play a role in lipid metabolism and gene regulation, as part of its mechanism of action. The paper underscores the intricate nature of fingolimod's impact on PPARs and CD36 and invites further research to understand its clinical efficacy.

The work by Portela-Lomba et al. probes the potential of pharmacological reprogramming of olfactory ensheathing glia into functional neurons. This paper highlights the challenges in achieving complete conversion of somatic cells into neurons using pharmacological approaches. It stresses the need for comprehensive strategies to enhance the efficiency of reprogramming techniques.

The review by Maciak et al. provides a comprehensive overview of the role of microRNA-mediated strategies in remyelination in MS. MicroRNAs, the orchestrators of gene expression, are pivotal in the remyelination process. This paper discusses their potential diagnostic and therapeutic applications, emphasizing their importance in the context of MS and other related disorders.

The final work by Braz et al. presents a compelling example of an innovative therapy in Krabbe disease, focusing on the neuroprotective effects of HDAC6 inhibition using ACY-738. This study effectively counters early neuropathological defects in Twitcher mice, hinting at the promise of ACY-738 as a neuroprotective therapy. This research provides valuable insights into potential treatment strategies for Krabbe disease and related conditions.

Collectively, these six papers offer a rich tapestry of knowledge and innovation in the pursuit of remyelination and neuroregeneration therapies. They highlight the complexity of the challenges in MS and related disorders, and the multipronged approach needed to address them effectively. Each paper serves as a stepping stone in this journey. They highlight the importance of exploring innovative drug targets and alternative therapeutic interventions, to meet the unmet needs of individuals affected by MS and related demyelinating disorders. They underscore the imperative of pushing the boundaries of our understanding, translating these findings into clinical applications, and advancing the cause of remyelination and neuroregeneration.

The journey is ongoing, and as we reflect on the insights gained from this Research Topic, we look forward to further investigations, collaborative efforts, and the translation of these findings into clinical applications, offering hope to patients who battle these formidable neurological disorders. Together, we remain resolute in our quest to find effective treatments and ultimately, a cure.

## Author contributions

AR: Conceptualization, Project administration, Supervision, Writing—original draft, Writing—review & editing. MV-E: Conceptualization, Project administration, Supervision, Writing—original draft, Writing—review & editing. SO'S: Conceptualization, Project administration, Supervision, Writing—original draft, Writing—review & editing.

